# Allelotyping of pooled DNA with 250 K SNP microarrays

**DOI:** 10.1186/1471-2164-8-77

**Published:** 2007-03-16

**Authors:** Stefan Wilkening, Bowang Chen, Michael Wirtenberger, Barbara Burwinkel, Asta Försti, Kari Hemminki, Federico Canzian

**Affiliations:** 1 Department of Molecular Genetic Epidemiology, German Cancer Research Center (DKFZ), 69120 Heidelberg, Germany; 2 Helmholtz University Group Molecular Epidemiology, German Cancer Research Center (DKFZ), 69120 Heidelberg, Germany; 3 Center for Family Medicine, Karolinska Institute, SE-14183 Huddinge, Sweden

## Abstract

**Background:**

Genotyping technologies for whole genome association studies are now available. To perform such studies to an affordable price, pooled DNA can be used. Recent studies have shown that GeneChip Human Mapping 10 K and 50 K arrays are suitable for the estimation of the allele frequency in pooled DNA. In the present study, we tested the accuracy of the 250 K Nsp array, which is part of the 500 K array set representing 500,568 SNPs. Furthermore, we compared different algorithms to estimate allele frequencies of pooled DNA.

**Results:**

We could confirm that the polynomial based probe specific correction (PPC) was the most accurate method for allele frequency estimation. However, a simple *k*-correction, using the relative allele signal (RAS) of heterozygous individuals, performed only slightly worse and provided results for more SNPs. Using four replicates of the 250 K array and the *k*-correction using heterozygous RAS values, we obtained results for 104.141 SNPs. The correlation between estimated and real allele frequency was 0.983 and the average error was 0.046, which was comparable to the results obtained with the 10 K array. Furthermore, we could show how the estimation accuracy depended on the SNP type (average error for A/T SNPs: 0.043 and for G/C SNPs: 0.052).

**Conclusion:**

The combination of DNA pooling and analysis of single nucleotide polymorphisms (SNPs) on high density microarrays is a promising tool for whole genome association studies.

## Background

To find new susceptibility loci for complex diseases on the human genome, a high number of case and control samples is required. An old approach with new perspective is the pooling of cases and controls. The larger the number of analyzed SNPs, the more striking are the advantages of a pooling study. With advanced microarray technology it is now possible to analyze SNPs throughout the whole genome. With the Human Mapping 500 K array set from Affymetrix and the BeadChips from Illumina, over 500,000 SNPs can be genotyped on two arrays. Different groups have tested the reliability of Affymetrix microarrays for pooling studies with either the 10 K array [1-6] or the 50 K array [7,8]. On these arrays, each SNP is interrogated by 40 probes (20 for the plus and 20 on the minus strand). On the 250 K arrays over 90% of the SNPs are represented by only 24 probes (some SNPs are only on the plus or the minus strand). This reduction of probes, as well as the reduction of the feature size from 18 μm (10 K), and 8 μm (50 K) to 5 μm (250 K) could have a negative influence on the outcome of pooling results. To examine if this is true, we tested the Nsp I 250 K array which represents 262.264 SNPs and is part of the 500 K array set. According to the Data Sheet from Affymetrix, over 85% of the human genome is covered by SNPs within 10 kb distance with this array set. If allelotyping of pooled DNA is feasible with these arrays, whole genome association studies including thousands of samples could be performed within a few weeks in a cost-effective manner.

## Results

### 10 K array

To assess the measurement error in our lab, we estimated the allele frequency in a pool of 26 DNA samples previously genotyped in our lab with the 10 K array. We calculated the allele frequency with three methods (see Material and Methods). As reference data for the correction of unequal allele signals, we took either data generated in our lab ("our") or data from other labs ("web" or "brohede"). From 10,561 SNPs on the 10 K array, the allele frequency of 3,574 SNPs could be estimated with all three methods. In Table 1, we show the mean and median error (absolute difference between known and estimated allele frequency), the correlation coefficient between known and estimated allele frequency, and the standard deviation (SD) between the four replicates. As expected, the estimates were better when using the reference data generated in our lab. The PPC method was the most accurate method with a mean error of 0.043. However, the k-correction with heterozygous RAS values gave only slightly worse results with an error of 0.046. In comparison with other methods the PPC is the only algorithm that uses only perfect match data. To elucidate if the k-correction can be improved by utilizing just perfect match data, we set all cell intensity values in the original cell files to zero. Then we derived a perfect-match-RAS and reanalyzed the data using the k-correction with heterozygous references. The resulting estimates gave an average error of 0.108. Applying a second degree polynomial on these perfect-match-RAS values could reduce the error to 0.054. However, for "normal" RAS values the second degree polynomial did not improve the error.

**Table 1:** Comparison of accuracies of three algorithms

method_source*	**mean error**	**median error**	**correlation**	**mean SD**
Simpson_our	0.046	0.034	0.951	0.051
Simpson_web	0.051	0.038	0.941	0.056
Craig_our	0.067	0.049	0.909	0.072
Craig_web	0.080	0.061	0.903	0.075
PPC_our	0.043	0.033	0.959	0.022
PPC_brohede	0.050	0.038	0.946	0.022

The errors are based on estimates from 3574 SNPs which could be analyzed by all methods.

*Data used for normalization: "our" = 34 individuals analyzed in our lab, "Brohede" = 26 individuals analyzed in the lab of Brohede et al. [1], "web" >3000 individuals analyzed in the lab of Caig et al. [9], files are available under [15].

### 250 K array

From the 262,264 SNPs on the Nsp 250 K array, the rs-numbers of 195,158 SNPs could be identified from the HapMap CEPH Population (NCBI_Build35). We excluded 137 SNPs (3 on Chr. 1, 128 on Chr. 2, 6 on Chr. 16) which had inconsistent genotype information in the two sources (e.g. rs1364648, Affymetrix annotation: A/G, minus-strand; HapMap data: C/G, plus-strand). From the remaining SNPs, 122,754 had a 100% call rate in the 88 HapMap samples. For the evaluation, 104,141 SNPs could be used because they had at least one "AB" genotype (required for *k*-correction) in the 56 reference samples genotyped in our lab. Table 2 shows the mean error, the correlation coefficient between known and estimated allele frequency, and the standard deviation between the pool replicates. We also specified how the accuracy depended on the number of pool replicates, the number of reference RAS values (with AB genotype), the minor allele frequency, and the SNP type. As expected, we found that the mean error decreased by the number of pool replicates. The mean error also decreased by the number of "AB" reference samples, and with an increasing minor allele frequency. To see if the error improves with higher allele frequencies only because of a higher number of "AB" references or vice versa, we adjusted both parameters and found the same trend. We could further show that the estimation of the allele frequency in A/T SNPs was significantly less accurate than in G/C SNPs (p < 0.001). The same trends were found for the 10 K array (results not shown).

**Table 2:** Estimation accuracy in the Nsp 250 K array

no. of pool replicates*	**mean error**	**SNPs**	**correlation**	**mean SD**
1	0.056	91647	0.971	0.000
2	0.051	93654	0.976	0.041
3	0.047	99922	0.980	0.044
4	0.046	102687	0.983	0.046

**no. of "AB" references**	**mean error**	**SNPs**	**correlation**	**mean SD**

1	0.095	4790	0.980	0.041
2	0.079	3544	0.987	0.041
3	0.070	3479	0.989	0.041
4	0.064	3523	0.989	0.042
5	0.061	3364	0.986	0.043
6	0.057	3623	0.989	0.043
7	0.054	3543	0.988	0.043
8	0.052	3356	0.987	0.045
9	0.049	3419	0.988	0.045
10	0.048	3524	0.987	0.046
15	0.042	3545	0.980	0.048
20	0.035	3701	0.976	0.048
25	0.030	3208	0.959	0.046
30	0.027	1329	0.941	0.043
35	0.024	141	0.954	0.041

**minor allele frequency**	**mean error**	**SNPs**	**correlation**	**mean SD**

0.0 – 0.1	0.096	27688	0.915	0.037
0.1 – 0.2	0.045	23875	0.983	0.043
0.2 – 0.3	0.038	18843	0.977	0.048
0.3 – 0.4	0.033	17339	0.953	0.051
0.4 – 0.5	0.030	15783	0.778	0.053

**SNP type**	**mean error**	**SNPs**	**correlation**	**mean SD**

A/T, T/A	0.052	6799	0.979	0.050
A/C, T/G	0.048	16056	0.982	0.046
A/G, T/C	0.045	69445	0.983	0.045
C/G, G/C	0.043	10387	0.981	0.045

*To get the error for different numbers of repeats, we took the mean of all possible combination of the four replicates. For 3 replicates for example we took the mean values of pool combinations 123, 124, 134, 234.

**Figure 1:**
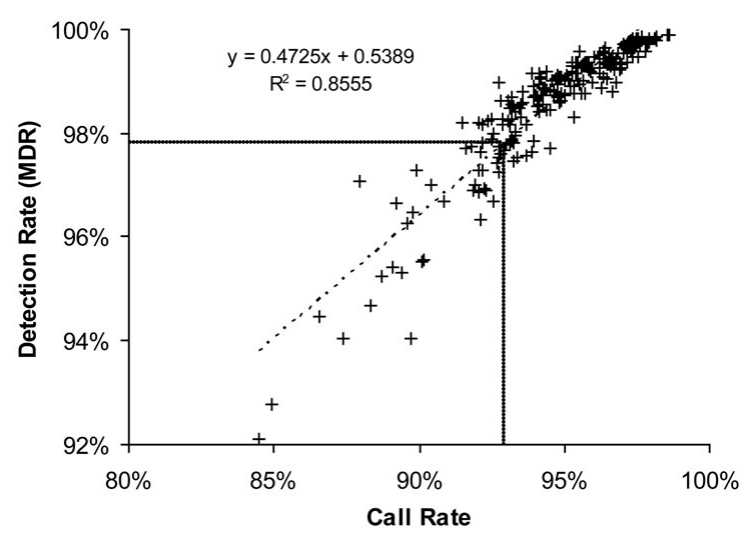
Graph showing the correlation between detection rate (MDR) and call rate. Data derived from 100 NspI and 100 StyI arrays, hybridized with individual DNA. A 93% call rate corresponds to about 97.8% MDR.

**Figure 2:**
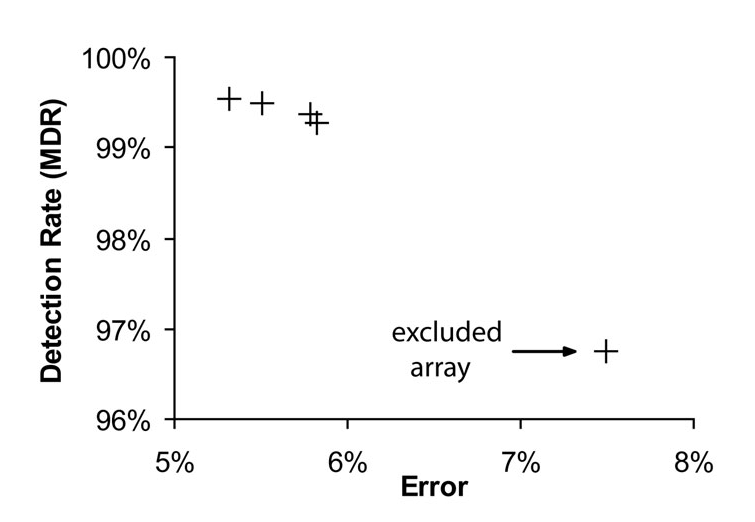
Graph showing the correlation between detection rate (MDR) and the error (absolute difference between estimated and known allele frequency). Each cross stands for one 250 K array, all hybridized with the same DNA pool.

For the reference samples, arrays with less than 93% call rate were excluded. For pooled DNA, however, the call rate normally is around 80%, because many SNP frequencies lie between homozygous and heterozygous frequencies. To prove if the call rate can be partially explained by the detection rate (MDR), we plotted the call rates against detection rates from 100 Nsp and 100 Sty arrays previously analyzed with individual DNA in our lab (Figure 1). According to the regression curve, a call rate of 93% corresponds to a detection rate of about 97.8%. One of our 250 K arrays (hybridized with pooled DNA) had a detection rate of 96.7%. It was therefore considered to be of bad quality and was excluded. This array also had a significantly poorer accuracy (error: 0.075). In the other four arrays (with MDR >99.2) a high MDR also correlated with a low error (see Figure 2).

## Discussion

With our data from the 10 K array, we could confirm that from the three tested methods, the PPC algorithm [1] gave the best estimates. Compared to other methods, this algorithm (a) utilizes the signal intensities from individual probes (not RAS values); (b) it takes only data from the perfect matches; (c) it applies a second degree polynomial for correction of unequal hybridization; and (d) it uses reference information from all three genotypes (AA, AB, BB). Our results suggest that neither of these parameters alone is responsible for the good performance of the PPC algorithm but the combination of all. However, the need for all three genotypes in the reference samples limits the number of SNPs that can be estimated. Another disadvantage of this method is the time consuming computation in Perl and R. This made it impossible to use the algorithm for our 250 K data yet. For the Nsp 250 K array, we used the *k*-correction with heterozygous RAS values. This algorithm performed only slightly worse than the PPC algorithm. It was the simplest of the tested algorithms and it scored for more SNPs, because homozygous calls were not required. The algorithm proposed by Craig et al. [9], also uses RAS values and includes reference information of all three genotypes, which should improve the estimation. However, this method gave the worst estimates for our data set. The algorithm used by Kirov et al. with a reported average error of only 0.014 with 10 K arrays might improve the allelotyping accuracy for 250 K arrays. Instead of using heterozygous references, the correction coefficient *k *is derived from RAS values of a pool with known allele frequencies. This algorithm was not applied here, because it requires a second independent DNA pool with known allele frequencies. Future studies can use our *k *values (supplied as Additional Material) for allele frequency estimation on the 250 K Nsp arrays. However, results for SNPs with a very low/high frequency in the reference pool may not be reliable. Another approach could be the combination of the PPC algorithm and the algorithm from Kirov et al. where *k *is calculated from pooled data of all perfectly matching probes. To avoid the use of reference data in a case-control study with pooled samples, it is also possible to directly compare the signal intensities of the perfectly matching probes between cases and controls as shown by Macgregor et al. [7]. In this study, the use of a correction for unequal hybridization signals had only little effect upon the results. However, also slight improvements can be important for the finding of low susceptibility genes in pooling studies.

Despite the reduction of the feature number and feature size, the absolute error between real and estimated allele frequency with the 250 K array was as low as the one for the 10 K array when using Simpson's *k*-correction. The correlation between real and estimated allele frequency was even higher with the 250 K array, and the standard deviation was lower. However, our results from the 10 K and the 250 K array are not directly comparable, because (a) pools were constructed from different DNA samples, (b) the experimental protocol was different, (c) different scanners were used for both chips, and (d) the software used for data extraction was different.

As shown in Table 2, the accuracy of the allele frequency estimation improved with the number of pool replicates. The absolute error between three and four replicates only decreased by 0.001. Therefore, we assume that the addition of further technical replicates would not essentially improve the accuracy. In our study, we used pools of identical samples. However, for a case-control study, it might be of advantage to use pools of independent samples to capture the variance between the individuals. In this case, an increase of replicates can improve the accuracy. With increasing number of "AB" references, the error decreased to 0.024 when 35 references were present. In our study, the mean error was smaller when the minor allele frequency was higher. This was also true for the 10 K results using the PPC algorithm, which is in contrast to the results published by Brohede et al. [1], where the best estimates were obtained at minor allele frequencies <0.1. Interestingly, the accuracy of A/T SNPs was found to be significantly worse than the accuracy of G/C SNPs on the 250 K array. This is probably due to the higher affinity of the G-C hydrogen bound compared to the A-T bound. For the stability of the entire hybridization complex, an unspecific hybridization with "A" or "T" is relatively less important than with "G" or "C". Here we analyzed only one of the two 250 K arrays from the 500 K set. The only difference between the two arrays is the cleavage side in the first fragmentation step. Therefore, we assume that both arrays, Nsp and Sty, perform equally well.

Pooling of samples has several disadvantages compared to a case-control study analyzing individual genotypes: (a) Associations which do not result in a significant change of the allele frequency can be overlooked; (b) Measurement errors can lead to false results; (c) Stratification of the population by age, sex, disease subtype, etc. has to be done before the analysis; (d) Haplotype analysis is only possible under certain conditions [10,11]; and (e) Analysis of gene-gene interactions can not be performed. However, with advancing technologies and algorithms, the mean measurement error can probably be reduced to values < 0.03 [1,4]. The use of linkage information should improve the likelihood of finding "real" associations and detect false positive SNPs. Taking the HapMap information (Build 35) for the 10 K array, we found ~30% of the SNPs to be linked to its downstream SNP (LOD >3); with the 500 K array set it was ~50%. With this high linkage, the allele frequency of one SNP can be partly explained by the allele frequency of a linked SNP. To take advantage of this fact, two recent publications propose to use p-value combinations in a sliding-window concept [9,12]. With increasing number of analyzed SNPs and better linkage information most haplotypes can be explained by individual SNPs [13].

## Conclusion

We think that DNA pooling might be a useful and affordable tool to detecting new candidate genes for genetic diseases, especially at a whole genome level. However, this has to be proven in future association studies with pooled DNA.

## Methods

### DNA pooling and microarray analysis

The determination of the DNA concentration in the individual DNA samples was done with PicoGreen reagent (Molecular Probes) using a standard curve of λ-DNA. From each sample, 50 ng genomic DNA was taken for the pool construction. For the 10 K array, we pooled 26 DNA samples that were individually genotyped before with the 10 K array. For the 250 K array we pooled 88 samples from the HapMap CEPH Population, whose genotype information is available at the HapMap homepage [14]. From individual or pooled samples 250 ng DNA was analyzed on the GeneChip Human Mapping 10 K Xba 131 array or the 250 K Nsp array (Affymetrix) according the manufacturers protocols. Four replicates of the same DNA pool from the 10 K and the 250 K array were processed and hybridized on four different days, respectively. Imaging of the microarrays was performed using either the GCS3000 scanner (10 K array) or the upgraded GCS3000-G7 scanner (250 K array) from Affymetrix. Genotype calls and probe intensity data were extracted with the GDAS software using default parameters (10 K array) or the GTYPE software from Affymetrix setting the call threshold for homozygous and heterozygous calls to 0.26 (250 K arrays). For individual DNA, only arrays with a call rate >93% (as guarantied by Affymetrix) were included in the study. For pooled DNA, only arrays with a detection rate (MDR) >97.8% (corresponding to call rate of >93%, see Results) were used for the allele frequency estimation. One array had to be repeated because of its low MDR (96.7%).

### Estimation of allele frequency with the 10 K array

On the 10 K array, each SNP is represented by 40 probes each 25 bp of length. The 40 probes are composed of 20 probes perfectly matching the SNP and 20 probes with a 1 bp mismatch. For the 10 K arrays, the analysis software from Affymetrix calculates the "Median Relative Allele Signal" for the forward (RAS1) and the reverse strand (RAS2) which are derived from all 40 probe intensities. Here, we compared three different algorithms, which take either the RAS values or the probe intensities from the 20 perfect matching probes as input. The *k*-correction proposed by Simpson, et al. uses RAS values (average of RAS1 and RAS2) from heterozygous genotypes [6]. The *k*-correction proposed by Craig et al. uses RAS values from all three genotypes [9]. For this correction we excluded RAS1 and RAS2 values with standard deviation >1 (SD from 4 pools) and set values <0 and >1 to 0 and 1, respectively. As reference data for the *k*-corrections (Simpson et al. and Craig et al.) we used RAS values from 34 arrays analyzed with individual DNA in our lab or RAS values from over 3000 arrays on the web page [15] provided by Craig et al. [9]. The polynomial based probe specific correction (PPC) from Brohede, et al. uses information of the individual perfect match probe pairs from all three genotypes [1]. As reference data for correction, we used 34 arrays previously analyzed in our lab or *k*-correction data from 26 arrays kindly provided by Jesper Brohede as external reference.

### Estimation of allele frequency with the 250 K array

For the 250 K arrays, the *k*-correction proposed by Simpson, et al. was used to estimate the allele frequencies [6]. Heterozygous RAS values were taken from a set of 56 arrays (all with call rates >93%), which were previously analyzed with individual DNA in our lab. The average RAS values as well as the discrimination scores were calculated from the cell intensity data using the "R" script from Meaburn et al. [8] which is freely available [16]. We excluded RAS values from the four pools which had discrimination scores < 0.04, as described by Meaburn et al [8]. The discrimination score (DS_snp _is a measure of unspecific hybridization used in the 10 K MPAM mapping algorithm (see Affymetrix GeneChip DNA Analysis Software users' guide for detailed information). This score ranges from 0 to 1 with higher scores indicating greater discrimination between perfect match probes and mismatch probes. Individual SNP data for *k*-correction is supplied as Additional Material, with *k *derived from heterozygous RAS values (see Additional file 1) and *k *derived from RAS values of pooled DNA (see Additional file 2).

## Authors' contributions

SW designed the study, constructed the DNA pools, and lead in drafting the manuscript. BC performed the statistical analysis. MW and BB performed most part of the microarray analysis. AF, KH, and FC contributed to interpretation of the data and the writing of the manuscript. All authors read and approved the final manuscript.

## Supplementary Material

Additional file 1 Heterozygous *k*: This table includes the SNP-ID, used by Affymetrix, the rs-number, *k *(deriving from heterozygous RAS values of 56 reference samples), the number of RAS values which were heterozygous, and the variation of these RAS values. *k *= RAS/(1-RAS), Simpson et al. 2005. Click here for file

Additional file 2 Pool *k*: This table includes the SNP-ID, used by Affymetrix, the rs-number, RAS values from pool 1 to 4, discrimination scores (DS), from pool 1 to 4, the average RAS from the four pools, the SD of the RAS values, the known frequency of allele A, and *k *(deriving from RAS values of pooled DNA). *k *= (RASpool-RASpool*Freq_A)/(Freq_A-RASpool*Freq_A), Kirov et al. 2006. Click here for file
